# Biomarkers for isolated congenital heart disease based on maternal amniotic fluid metabolomics analysis

**DOI:** 10.1186/s12872-022-02912-2

**Published:** 2022-11-20

**Authors:** Xuelian Yuan, Lu Li, Hong Kang, Meixian Wang, Jing Zeng, Yanfang Lei, Nana Li, Ping Yu, Xiaohong Li, Zhen Liu

**Affiliations:** 1 National Center for Birth Defect Monitoring, Key Laboratory of Birth Defects and Related Diseases of Women and Children, West China Second University Hospital, Sichuan University, Chengdu, Sichuan China; 2 Development and Related Diseases of Women and Children Key Laboratory of Sichuan Province, Sec.3 No.17, South RenMin Road, Chengdu, Sichuan China; 3 Department of Obstetrics & Gynecology, Longchang Maternal and Child Healthcare Hospital, Neijiang, Sichuan China; 4 Department of Obstetrics, Zhaotong Second People’s Hospital, Zhaotong, Yunnan China

**Keywords:** Congenital heart disease, Metabolomics, Reproduction, Biomarker, Diagnosis

## Abstract

**Introduction:**

Congenital heart disease (CHD) is one of the most prevalent birth defects in the world. The pathogenesis of CHD is complex and unclear. With the development of metabolomics technology, variations in metabolites may provide new clues about the causes of CHD and may serve as a biomarker during pregnancy.

**Methods:**

Sixty-five amniotic fluid samples (28 cases and 37 controls) during the second and third trimesters were utilized in this study. The metabolomics of CHD and normal fetuses were analyzed by untargeted metabolomics technology. Differential comparison and randomForest were used to screen metabolic biomarkers.

**Results:**

A total of 2472 metabolites were detected, and they were distributed differentially between the cases and controls. Setting the selection criteria of fold change (FC) ≥ 2, *P* value < 0.01 and variable importance for the projection (VIP) ≥ 1.5, we screened 118 differential metabolites. Within the prediction model by random forest, PE(MonoMe(11,5)/MonoMe(13,5)), N-feruloylserotonin and 2,6-di-tert-butylbenzoquinone showed good prediction effects. Differential metabolites were mainly concentrated in aldosterone synthesis and secretion, drug metabolism, nicotinate and nicotinamide metabolism pathways, which may be related to the occurrence and development of CHD.

**Conclusion:**

This study provides a new database of CHD metabolic biomarkers and mechanistic research. These results need to be further verified in larger samples.

**Supplementary Information:**

The online version contains supplementary material available at 10.1186/s12872-022-02912-2.

## Key points

What is already known about this topic?

The pathogenesis of congenital heart disease (CHD) is complex and unclear. Metabolomics could detect the changes in metabolites produced by biological systems, which may provide a new clue for CHD occurrence and serve as a biomarker for diagnosis.

What does this study add?

Metabolites in maternal amniotic fluid were distributed differentially between the CHD cases and controls. Differential metabolites may serve as screening biomarkers. They were mainly concentrated in three key pathways.

## Introduction

Congenital heart disease (CHD) is a structural abnormality caused by the formation or abnormal development of the heart and large blood vessels during embryonic development. CHD is one of the most prevalent of birth defects in the world [1]. The worldwide prevalence of CHD in live births was rising during the previous 40 years and reached 9.41‰ during 2010–2017 [1]. The etiologies of CHD occurrence are complex. It is reported that less than 30% of CHDs are caused by clear environmental or genetic factors, and the unknown causes are considered to be the result of interactions between environmental and genetic factors [2, 3]. However, most of the influencing factors have not been fully confirmed utill now, and the teratogenic mechanism is still lacks sufficient evidence, which seriously restricts the formulation and implementation of effective interventions for CHD. The cause and etiological mechanism of congenital heart disease have been studied for decades, but there is no obvious breakthrough providing a clear etiological mechanism. It is urgent to explore new ways to research the etiology of CHD from new perspectives and directions.

The heart is the first functional organ developed in the embryo. The primitive heart tube begins to form in the 3rd week of the embryo and plays a role in circulation in the 4th week. By the 8th week, the atrial and ventricular septa are fully grown, and the connection of the fundus arteries and veins is completed [4]. If it was affected by any teratogenic factors during this period, there may be abnormal development of the heart or large blood vessels.

Currently, the diagnosis of congenital heart disease is mainly concentrated in the second trimester, using ultrasound echocardiography. [5] However, the precision of CHD diagnosis is greatly limited by ultrasound technology in different hospitals [6, 7], and echocardiography itself has some limitations [6, 8]. Some mild CHD subtypes, such as isolated coarctation of the aorta (CoA), could not be identified until after birth [9]. Furthermore, CHD is not constant during pregnancy, and it may be dynamically change. For example, up to 60–80% of ventricular septal defects (VSD) can spontaneously recover [10, 11]. Therefore, whether a relatively objective inspection method could be developed to increase the prediction effect of CHD in later development needs be considered.

Metabolomics can quantitatively detect the changes in all metabolites produced by biological systems (cells, tissues or organisms) after external stimuli or genetic modification [12]. Metabolomics can discover disease-related small molecule compounds by studying the internal changes of biological systems. Understanding disease-specific metabolites provides a theoretical basis for exploring the pathogenesis of maternal–fetal diseases and searching for disease-related biomarkers [13, 14]. Metabolomics has been used in studies of congenital heart disease in recent years [15, 16]. The variable metabolites could provide important clues for exploring the pathogenesis and biomarkers of CHD.

Previous studies have used biological samples such as maternal serum [17], urine [18, 19], and amniotic fluid (AF) [20] to detect metabolites for exploring biomarkers of congenital heart disease. However, the types of detectable metabolites are limited. The results are inconsistent, and the possible mechanism remains unclear. In this study, amniotic fluid in the second and third trimesters was used as the research material, and a new metabolomic research method involving untargeted metabolomic assays was used to analyze the specific metabolic markers in AF. At the same time, we explored the metabolic mechanism of congenital heart disease to provide more research evidence.

## Materials and methods

### Study population and sampling

This project was based on a multicenter hospital-based case–control study. Pregnant women who underwent prenatal diagnosis during the second trimester were recruited from three hospitals in China [21]. Mothers whose fetuses were prenatally diagnosed with CHDs or without any anomalies were initially chosen as the cases and the controls, respectively. The phenotype was diagnosed by sonographers, pathologists, and pediatricians through systematic ultrasound, autopsy, or postnatal follow-up [21, 22]. Cases were defined as isolated congenital heart disease without other extracardiac malformations, and controls were defined as fetuses without any congenital anomalies.

A structured questionnaire-based interview was used to collect the subjects’ information after the pregnant women were signed informed consent [23, 24]. During the follow-up process, when the subjects needed to undergo amniocentesis for medical reasons, 5 ml of amniotic fluid was extracted in the amniocentesis procedure with the woman’s consent. The amniotic fluid was centrifuged, and the supernatant was stored in aliquots at -70 °C until analysis. This study was approved by the Medical Ethics Committee of Sichuan University (ID: 2,010,004) and West China Second University Hospital (ID: 2015(011)). All subjects provided informed consent to participate. Here, all samples and information were obtained from the project biobank. The subtypes of CHD cases are listed in Table 1. Both cases and controls were singletons without a family history of CHD.

**Table 1:** The subtypes of CHD cases

CHD cases	ICD-10	Number
VSD	Q21.0	6
TOF	Q21.3	10
AVSD	Q21.2	2
SV	Q20.4	3
SA,SV	Q21.2;Q20.4	2
PTA,VSD	Q20.0;Q21.0	2
TGA,PA,VSD	Q20.3;Q21.0; Q25.5	1
RAI	Q20.6	1
LA-RVF,PVM	Q22.3;Q24.5	1
Total		28

*VSD* Ventricular septal defects, *TOF* Tetralogy of fallot, *AVSD* Atrioventricular septal defect, *RAI* Right atrial isomerism, *SV* Single ventricle, *SA* Single atrium, *PTA* Persistent truncus arteriosus, *TGA* Transposition of the great arteries, *PA* Pulmonary atresia, *LA-RVF* Left coronary—right ventricular fistula, *PVM* Pulmonary valve malformation

### Sample pretreatment

A 100 μL sample of Amniotic fluid supernatant was transferred to an EP tube, and added 400 μL of extraction solution (acetonitrile: methanol = 1: 1) (CNW Technologies) containing isotopically labeled internal standard mixture was added. After vortexing, sonication, ice-water bath, incubated and centrifugation, 400μL of supernatant was transferred to a fresh glass vial and dried in a vacuum concentrator at 37 ℃. Then, the dried samples were reconstituted in 200 μL of 50% acetonitrile, and the above steps repeated. 75 μL of supernatant was transferred to a fresh glass vial for LC/MS analysis. The quality control (QC) sample was prepared by mixing an equal aliquot of the supernatants from all of the samples.

### Metabolomic measurements

Ultra (high) performance liquid chromatography (UHPLC) separation was carried out using an ExionLC Infinity series UHPLC System (AB Sciex), equipped with a UPLC BEH Amide column (2.1 × 100 mm, 1.7 μm, Waters). The mobile phase consisted of 25 mmol/L ammonium acetate and 25 mmol/L ammonia hydroxide ( CNW Technologies) in water (pH = 9.75) (A) and acetonitrile (B). The analysis was carried out with an elution gradient. The column temperature was 25℃. The autosampler temperature was 4 ℃, and the injection volume was 2 μL (pos) or 2 μL (neg), respectively.

TripleTOF 5600 mass spectrometry (AB Sciex) was used for its ability to acquire MS/MS spectra on an information-dependent basis (IDA) during LC/MS experiments. In this mode, the acquisition software (Analyst TF 1.7, AB Sciex) continuously evaluates the full scan survey MS data as it collects and triggers the acquisition of MS/MS spectra depending on preselected criteria. In each cycle, the most intensive 12 precursor ions with intensity above 100 were chosen for MS/MS at a collision energy (CE) of 30 eV. In the detection process, standard products and blank controls were strictly used for quality control, and data quality control was also carried out. The UHPLC-QTOF-MS analysis was performed at Biomarker Technologies Corporation, Beijing, China (https://international.biocloud.net/).

### Statistical analysis

#### Descriptive statistics

A case–control analysis was performed to assess the variations in metabolites on CHDs. The personalized features included maternal age, maternal prepregnancy body mass index (ppBMI), fetal sex (male, female), and gravidity. Frequency was used to describe qualitative data, and continuous variables were described as quantitative data using the mean and SD. Differences in the frequencies of these factors between cases and controls were assessed using the chi-square test or Student’s t-test.

#### Metabolomics data processing

Mass spectrometry (MS) raw data (.wiff) files were converted to the mzXML format by ProteoWizard. The process including peak deconvolution, alignment and integration, was processed by R package XCMS (version 3.2). Minfrac and cut off are set as 0.5 and 0.3 respectively. In-house MS2 database was applied for metabolite identification.

Metabolomics data were analyzed using SIMCA-P14.0 (Umetrics, Umea, Sweden) software for differentially grouped principal component analysis (PCA), fold change (FC) analysis, orthogonal partial least squares discriminant analysis (OPLS-DA) and unsupervised clustering analysis. Dimensionality reduction and sorting of metabolites were performed to screen for differential metabolites. The methods of combining the multiple of FC, the *P* value of the Wilcoxon-Mann–Whitney rank sum test, the variable importance for the projection (VIP) value of the OPLS-DA model and the multivariate logistic regression analysis were used to screen the differential metabolites and mapping.

#### Metabolic biomarker screening

The random forest (RF) method was used to select the most influential markers. RF was implemented using the ‘randomForest’ function from the ‘randomForest’ package in R [25]. In addition, a receiver operating characteristic (ROC) curve was used to estimate the area under the curve (AUC) score and 95% confidence interval (95% CI) of each selected marker. A logistic regression model including these selected markers was conducted, and the Akaike information criterion (AIC), Bayesian information criterion (BIC), Positive Predictive Value (PPV), Negative Predictive Value (NPV), and AUC score were used to estimate the performance of the model for classifying the subjects. Additionally, sensitivity analysis was conducted to evaluate the robustness of the model.

#### Metabolic pathway analysis

The classification information of differential metabolites was annotated by using the HMDB (Human Metabolome Database, https://hmdb.ca/) and KEGG ((Kyoto Encyclopedia of Genes and Genomes, https://www.kegg.jp/) databases. Enrichment analysis and statistical drawing of the annotated differential metabolites were performed.

All analyses were carried out using R version 3.6.1 (R Foundation for Statistical Computing, http://www.r-project.org). Two-tailed values of *P* < 0.05 were considered significant.

## Results

### Major characteristics of the participants

After applying the inclusion and exclusion criteria to subjects with qualified biological samples, 65 amniotic fluid samples (28 cases and 37 controls) were ultimately recruited in the present study.

The demographic characteristics are listed in Table 2 and the individual information of the 28 CHDs are listed in Appendix Table S1. The proportions of gestational age and fetal sex were significantly different between the case and control groups (*p* < 0.05) (Table 2).

**Table 2:** Comparison of demographic characteristics between the two groups

	CHDs	Controls	χ^2^/T	*P* value
Number of samples	28	37		
Age, mean (SD)	28.0(4.1)	28.2(6.1)	0.121	0.90
Gestational age (week), mean (SD)	27.0(5.1)	32.2(8.7)	2.81	0.01
ppBMI, mean (SD)	20.2(3.0)	20.2(1.9)	-0.044	0.96
Fetal gender, n(%)				
male	10(35.7)	23(62.2)	4.461	0.04
female	18(64.3)	14(37.8)		
Gravidity, mean (SD)	1.97(1.24)	2.04(1.35)	-0.195	0.85
Hospital, n(%)				
Shenzhen	10(35.7)	8(21.6)	4.769	0.09
Guangxi	9(32.1)	7(18.9)		
Fujian	9(32.1)	22(59.5)		
Residence, n(%)				
City	16(57.1)	26(70.3)	1.201	0.273
Subway	12(42.9)	11(29.7)		
Factory nearby, n(%)				
Yes	6(21.4)	4(10.8)	1.380	0.24
No	22(78.6)	33(89.2)		
Farmer, n(%)				
Yes	1(3.6)	1(2.7)	0.04	0.841
No	27(96.4)	36(97.3)		
Secondhand tobacco, n(%)				
Yes	11(39.3)	18(48.6)	0.565	0.452
No	17(60.7)	19(51.4)		
Folate supplement, n(%)				
Yes	25(89.3)	29(78.4)	1.349	0.246
No	3(10.7)	8(21.6)		

### Metabolomics detection results

**Fig. 1:**
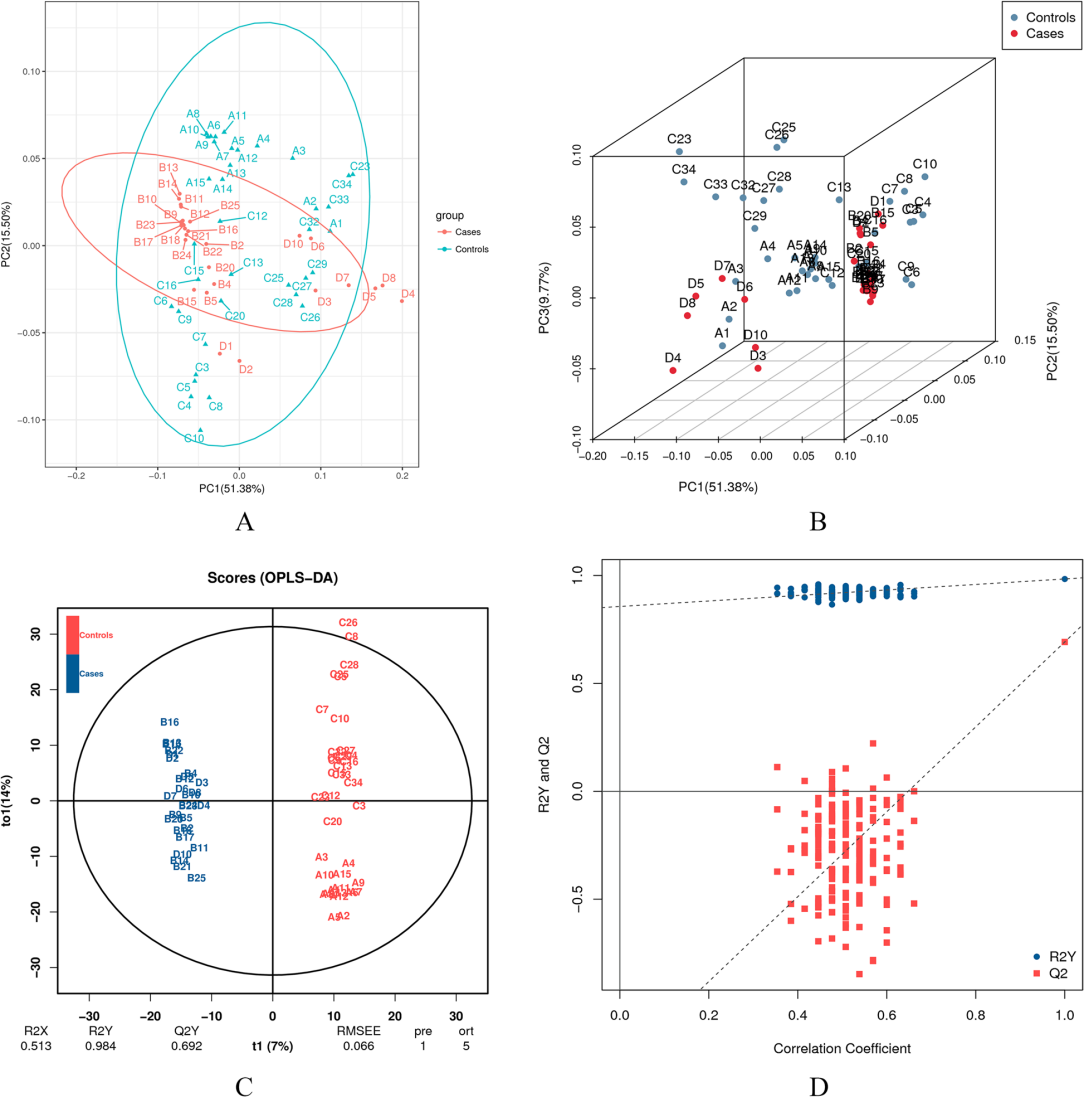
Metabolomics analysis between the two groups. **A**, PCA of all samples, where the X-axis represents the first principal component and the Y-axis represents the second principal component. **B**, PCA 3D map of differential grouping. **C**, OPLS-DA score map. **D**, OPLS-DA model validation diagram

Sixty-five samples were subjected to untargeted metabolomic assays, and a total of 2472 metabolites were identified. All metabolites were evaluated by principal component analysis (PCA), sample cluster analysis and repeated correlation evaluation. The distribution of metabolites between the case and control groups is shown in Fig. 1A, Band Figure S1A/B.

Orthogonal projections to latent structures-discriminant analysis (OPLS-DA) of differential groups of cases and controls was performed to obtain more reliable information on the degree of correlation between group differences in metabolites and experimental groups. In this model, R2Y was 0.984, and Q2 was 0.692. The results were shown inFig. 1C, D. The OPLS-DA results showed that there was a significant difference in metabolic profiles between CHD patients and controls in AF.

## Biomarker screening and validation

### Differential metabolite screening

For subjects with biological replicates, the method of combining the fold change (FC), the P value of the t-test and the VIP value of the OPLS-DA model was used to screen the differential metabolites. The screening criteria were FC > 2, P value < 0.01, and VIP > 1.50. A total of 118 differential metabolites were screened, of which 59 were upregulated and 59 were downregulated (listed in Table S2). The results are shown with the volcano map in Figure S1C.

## Results of randomforest

The more values below the detection limit, the less accurate the representation of the metabolite may be. Hence, we selected the candidate markers, with a detection value of 0 in less than 10% of subjects and 36 markers were selected from the 118 markers. Then, we selected 10 markers with an average VIP ≥ 1.8 as the most influential markers for the following analysis.

**Fig. 2:**
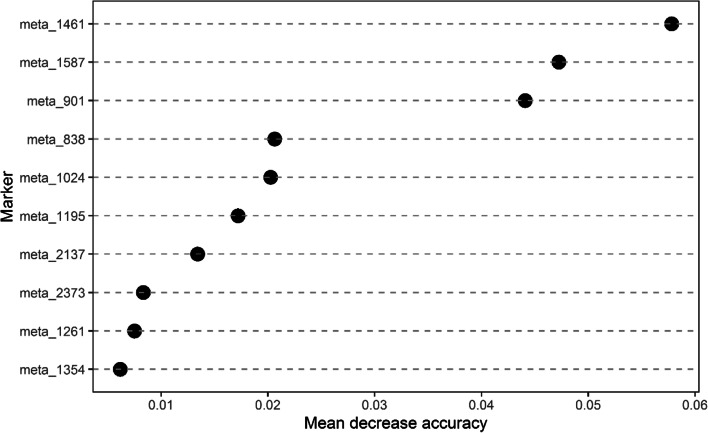
The 10 features ranked by mean decrease accuracy for the CHD random forest model. meta_1461: PE(MonoMe(11,5)/MonoMe(13,5)) meta_1587: 4-[N-(p-Coumaroyl)serotonin-4’’-yl]-N-feruloylserotonin meta_901: 2,6-Di-tert-butylbenzoquinone meta_838: 3-Methylglutarylcarnitinemeta_1024: Mytilin B meta_1195: N-[(4E,8Z)-1,3-dihydroxyoctadeca-4,8-dien-2-yl] hexadecanamide 1-glucoside meta_2137: Medicagenic acid 28-O- [b-D-xylosyl-(1- > 4)-a-L-rhamnosyl-(1- > 2)-a-L-arabinosyl] ester meta_2373: C16:1-OH Sphingomyelin (SM(d18:0/16:1(9Z)(OH))) meta_1261: Glabrolide meta_1354: Lysophosphatidylcholine acyl C 10:0 (LysoPC10:0)

When the number of decision trees (ntree) was 38, and the number of variables contained in each decision tree (mtry) was 9, the out-of-bag error was minor and tended to stabilize (Figure S2, Figure S3). As is shown in Fig. 2, meta_1461 emerged as the most important input variable, followed by meta_1587 and meta_901 (all of the above markers have a Mean Decrease Accuracy > 4.0). Less important variables included meta_838, meta_1024, meta_1195, meta_2137, meta_2373, meta_1261, and meta_1354 (all of these markers have a Mean Decrease Accuracy < 4.0). We used a logistic regression model to assess the performance of the combination of these markers whose Mean Decrease Accuracy > 4.0 for predicting the class of the subjects. The confusion matrix is shown in Figure S4.

**Fig. 3:**
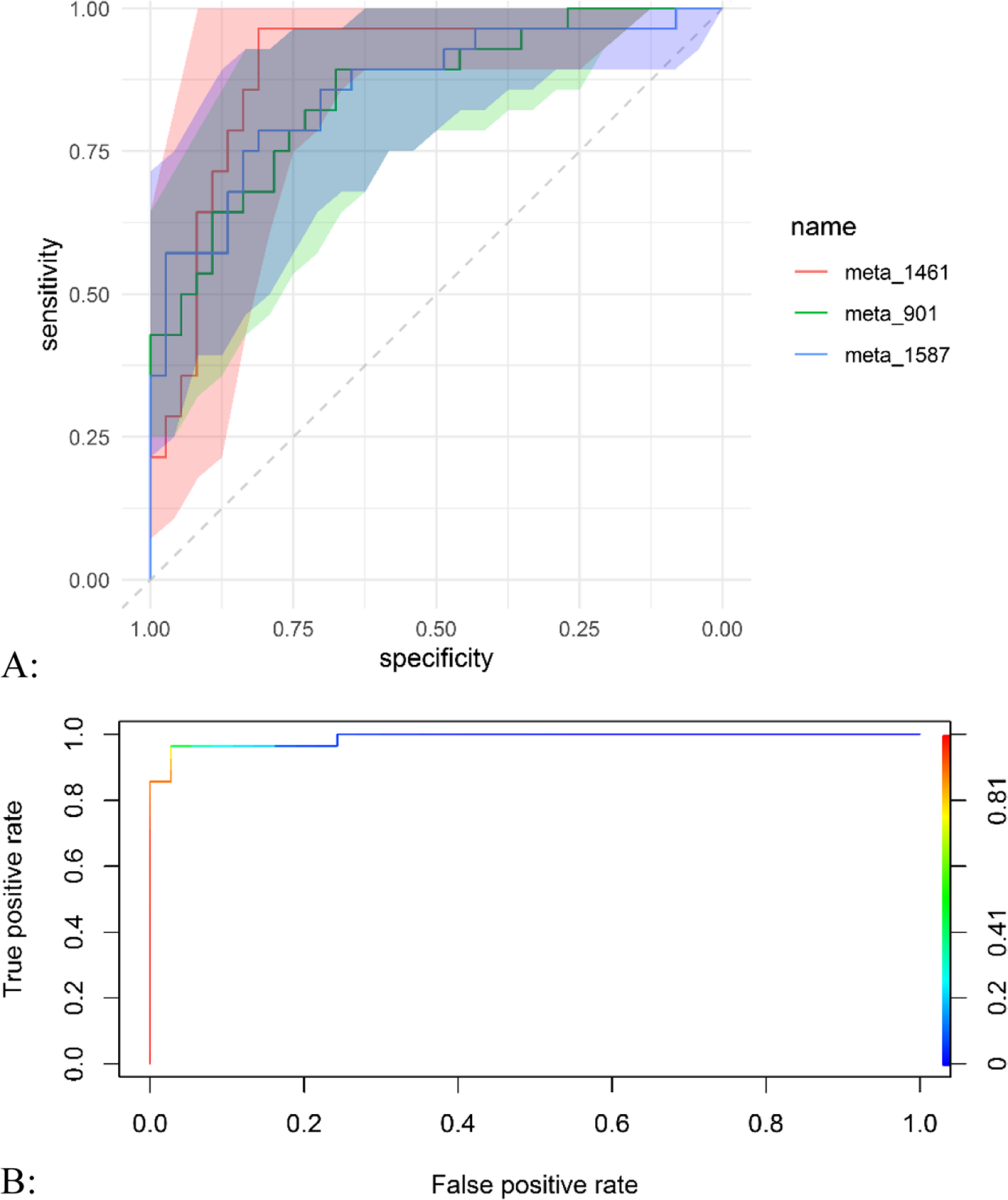
The ROC curves for classifying the subjects. **A**, The ROC curves comparing the performances of meta_1461, meta_901 and meta_1587. **B**, The ROC curve of the logistic regression model for classifying the subjects

## Performance of the selected markers

The ROC curve is the plot of the True Positive Rate (TPR) against the True Negative Rate (TNR) at varying classification thresholds. The ROC curves compared the performances of meta_1461, meta_901 and meta_1587 for classifying the subjects. It showed that meta_1461 performed best, with an AUC score of 89.6% (95% CI: 81.4%, 97.8%) (Fig. 3A). The meta_1587 ranked second (AUC score: 85.6%, 95% CI: 76.1%, 95.2%), followed by the meta_901 (AUC score: 85.0%, 95% CI: 75.7%, 94.4%) (Fig. 3A).

The ROC curve of the model is shown in Fig. 3B, with an AUC score of 98.8%. Furthermore we also performed a sensitivity analysis with some covariates in the logistic regression model, such as: maternal age, maternal prepregnancy Body Mass Index (ppBMI), fetal sex (boy, girl), and the number of pregnancy times. The AUC score (99.5%) of the adjusted model (with covariates adjusted in the model) was similar to that (98.8%) of the model without any covariates adjusted. The AIC decreased from 25.15 to 23.68, and the BIC increased from 33.85 to 38.90 (Fig. 3B).

**Fig. 4:**
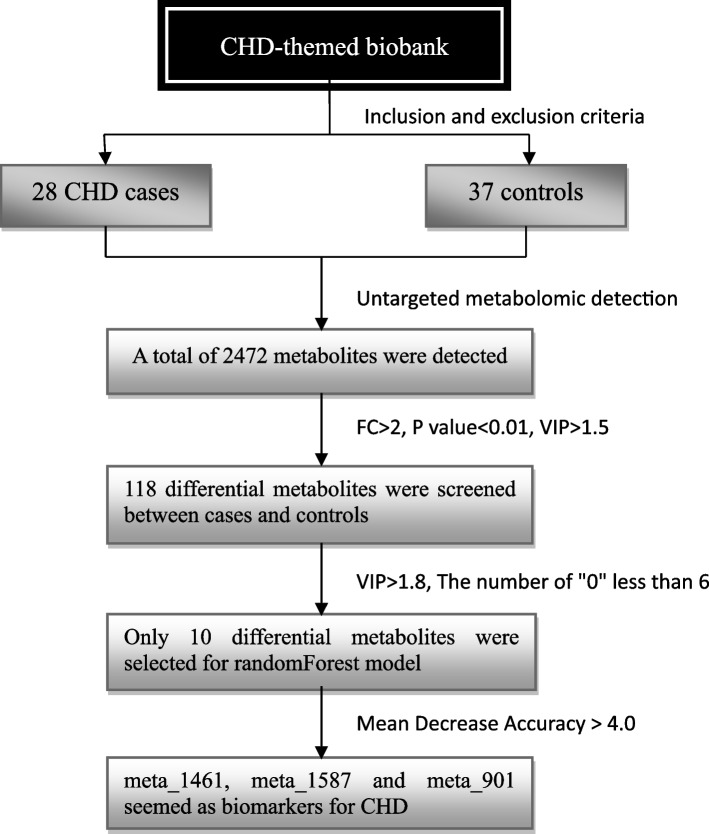
The process and results for metabolite biomarker screening

In summary, the metabolite biomarker screening process and results were showed in Fig. 4.

**Fig. 5:**
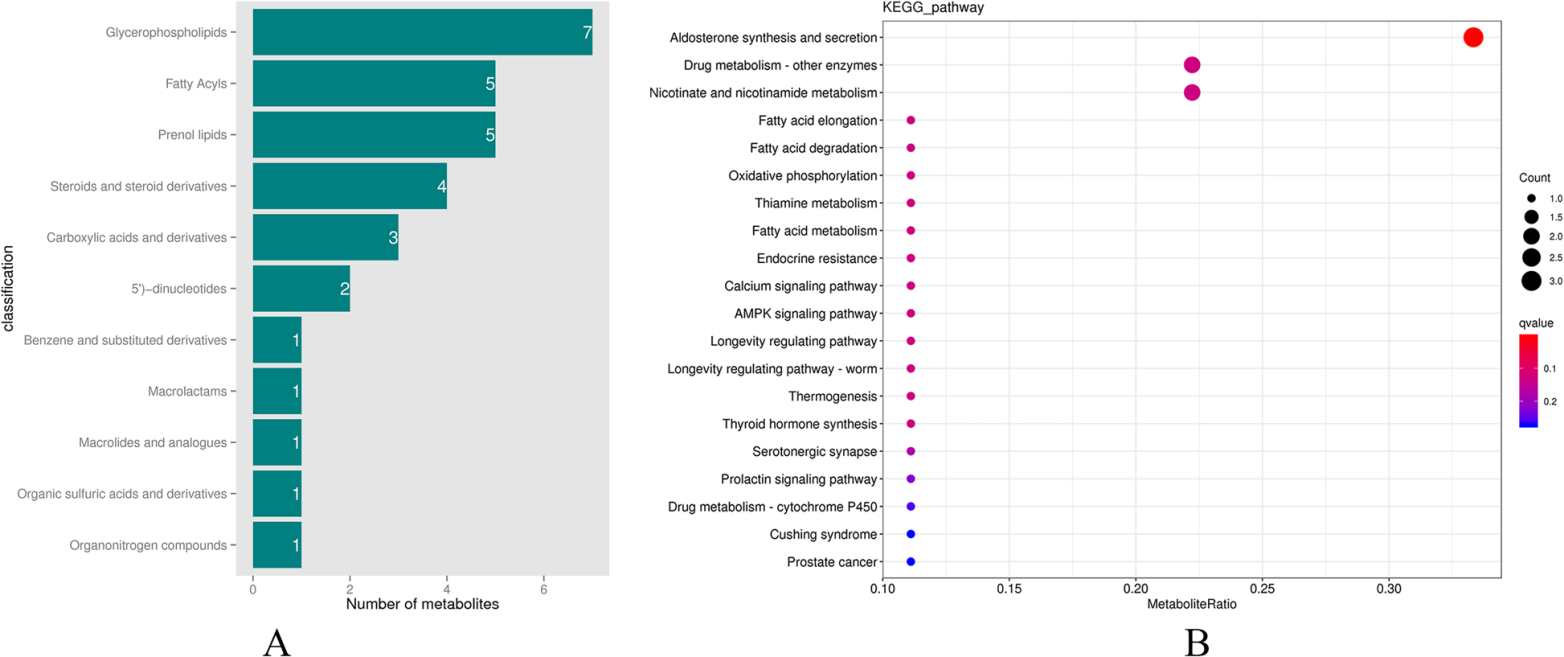
Differential metabolite enrichment pathways. **A** HMDB classification map of the differential metabolites in each group. **B** KEGG enrichment map of the differential metabolites

### Metabolic pathway analysis

Differential metabolites were annotated by the KEGG (Kyoto Encyclopedia of Genes and Genomes) database and HMDB (Human Metabolome Database). The hypergeometric test was used in ClusterProfiler to perform enrichment analysis on the annotation results of the KEGG differential metabolites [26]. The results showed that the differential metabolites were mainly concentrated in glycerophospholipids, fatty acyls and prenol lipids (Fig. 5A). These metabolites were enriched in aldosterone synthesis and secretion, drug metabolism, nicotinate and nicotinamide metabolism pathways (Fig. 5B).

## Discussion

In this study, the distribution characteristics of amniotic fluid metabolites in CHDs and the controls were obtained through untargeted metabolomics detection. Possible biomarkers for CHD occurrence or development were screened. We also explored the possible mechanisms for differential metabolites in the occurrence of CHD. Our results provide basic data resources into congenital heart disease from a new perspective.

Congenital heart disease is one of the most common birth defects [1], and the diagnosis of the disease is overly dependent on the technical level of ultrasonography [5].

Metabolomics is an extension of genomics that can more intuitively reflect the profiling of metabolites in biofluids, cells and tissues and it is routinely applied as a tool for biomarker discovery [27]. Owing to innovative developments in informatics and analytical technologies and the integration of orthogonal biological approaches, it has become possible to expand metabolomic analyses to understand the systems-level effects of metabolites, which can be used for CHD screening or to explore the mechanism of occurrence and development of CHD.

A previousstudy conducted a serum metabolomics study on children with congenital heart disease and found that 13 metabolites showed a significant increasing or decreasing trend. Taurine, glutamine, and glutamate presented considerable diagnostic value for the diagnosis of CHD [16]. Some researchers performed metabolomics detection on the serum of patients with congenital bicuspid aortic valve (BAV) and controls [15]. A predictive model for estimating group BAV was established and those studies supported the value of serum-based metabolomic profiling methods as an adjunct tool for screening large populations.

However, these studies used infant or childhood serum samples as material to explore the relationship between metabolites and CHD, and it is relatively rare to study the occurrence of CHD through biological samples obtained from pregnant mothers. Previous studies reported that maternal serum [17], urine [18, 19], and amniotic fluid (AF) [20] were used to detect metabolites by [1] H NMR or GC–MS technology. However, these methods for detecting metabolites are more limited than those detected by UHPLC‒MS.

Amniotic fluid, as the growing environment of the fetus, is relatively stable in the middle and late pregnancy stages. Compared with maternal blood, urine and other samples, it can better reflect the actual metabolic state of the fetus. The urine excreted by the fetus after the second trimester is an important source of amniotic fluid, and the metabolites in the fetus will be reflected in the amniotic fluid with the excretion of urine. The sources of amniotic fluid in the second and third trimesters are basically similar. Amniotic fluid not only provides a mechanical buffer for the fetus to prevent limb adhesion but also provides nutrients and growth factors, transports metabolites, and more. At the same time, the physiological and biochemical levels of the amniotic fluid reflect the health status of the fetus. Accurate and sensitive details of birth defect-related metabolites and their respective biochemical pathways can be obtained through amniotic fluid metabolomics, which also allows a better understanding of the overall pathophysiology of affected pregnancies.

A total of 2472 metabolites were identified using the UHPLC-QTOF-MS untargeted metabolomics detection in this study. Many new metabolites were found compared to previous studies, which mostly used NMR or GC-TOF–MS methods, and could only detect hundreds of metabolites [17–20]. UHPLC is increasingly displacing conventional high performance liquid chromatography [28] LC–MS is the main workhorse of metabolomics owing to its high degree of analytical sensitivity and specificity when measuring diverse chemistry in complex biological samples [29]. The untargeted metabolomics detection method can identify as many metabolites as possible by comparing characteristic peak ions with standard databases, and useing semiquantitative metabolite content to obtain high-throughput metabolomics data [28]. Untargeted metabolomics is a powerful tool that can provide new clues for prenatal diagnosis [14]。It will be helpful for discovering affected metabolic pathways, revealing disease pathogenesis, and identifying potential biomarkers [27].

The method of combining the fold change, the P value of the t-test and the VIP value of the OPLS-DA model was utilized to screen the differential metabolites, and the machine algorithm of randomForest (RF) was exploited to screen the biomarkers. The randomForest is an ensemble learning method that operates by constructing a collection of decision trees [30], and for variable selection, it performs well across sample sizes [31]. In addition, a receiver operating characteristic (ROC) curve was used to estimate the area under the curve (AUC) score and 95% confidence interval (95% CI) of each selected marker. We also evaluated the combined differentuation ability of these makers using a logistic regression model. The results show that PE(MonoMe(11,5)/MonoMe(13,5)), 4-[N-(p-Coumaroyl) serotonin-4’’-yl] -N-feruloylserotonin and 2,6-Di-tert-butylbenzoquinone in maternal amniotic fluid perform well in distinguishing cases from controls.

PE(MonoMe(11,5)/MonoMe(13,5)), also called 13-(3-methyl-5-pentylfuran-2-yl) tridecanoate, a kind of dimethylfuran fatty acid, is abundant in fish oil and is easily oxidized and degraded [32]. There are few reports about this chemical, but it has been found to be decreased in patients with gastrointestinal diseases [33]. 4-[N-(p-Coumaroyl)serotonin-4’’-yl]-N-feruloylserotonin is a serotonin derivative with a trace distribution in medicinal plants such as safflower [23]. It has strong scavenging free radicals and anti-lipid peroxidation ability, antitumor activity, anti-inflammatory and bacteriostatic effects, and it inhibits the production of melanin and other functional activities. This substance has the potential for the study of atherosclerosis and aortic wall distention [24]. 2,6-Di-tert-butylbenzoquinone, a cyclic NIAS originating from food packaging, has not been found to be associated with disease occurrence. However, a similar substance 2,5-di-(tert-butyl)-1,4-benzohydroquinone, is a reversible inhibitor of cardiac cells through intracellular Ca^2+^ handling in ventricular myocytes [34]. Among the ten most important metabolites, methylglutarylcarnitine was also reported detected differentially in CHD patients and controls [17]. Deficiency of 3- methylglutarylcarnitine affects the metabolism of leucine as well as ketogenesis. This disorder is one of an increasing list of inborn errors of metabolism that present clinically, such as metabolic syndrome (MetS), risk of developing cardiovascular disease (CVD) and type 2 diabetes [35].

This study found that the differential metabolites were mainly concentrated in several metabolic pathways, and it was inferred that aldosterone synthesis, drug metabolism, nicotinate and nicotinamide metabolism played very important roles in the occurrence and development of CHD. The secretion of aldosterone is mainly regulated by renin-angiotensin, a hormone that regulates the blood volume in the human body. It maintains water balance by regulating the reabsorption of sodium in kidneys. Excessive circulating and tissue angiotensin II (AngII) and aldosterone levels lead to a profibrotic, proinflammatory, and hypertrophic milieu [36] that causes remodeling and dysfunction of cardiovascular and renal tissues [37]. Nicotinate and nicotinamide are collectively referred to as vitamin 22. Nicotinamide forms coenzyme I and coenzyme II with ribose, phosphate and adenine in the body. They are the coenzymes of many dehydrogenases and are associated with many metabolic processes including glucose glycolysis, fat metabolism, and pyruvate metabolism, which are closely related to the formation of high-energy phosphate bonds [38]. As the major coenzyme in fuel oxidation and oxidative phosphorylation and a substrate for enzyme responses to energy stress and oxidative stress, nicotinamide adenine dinucleotide (NAD +) is emerging as a metabolic target in a number of diseases including heart failure. Niacin turns into niacinamide in the body to play the above role. In addition, niacin also has a strong peripheral vasodilator effect. Nicotinamide adenine dinucleotide (NAD) is synthesized de novo from tryptophan through the kynurenine pathway. The patients showed treduced levels of circulating NAD. Defects similar to those in the patients developed in the embryos of Haao-null or Kynu-null mice owing to NAD deficiency. The prevention of NAD deficiency during gestation could prevent these defects [39]. These results would provide additional new metabolite data sources for the CHD, and suggest a new idea for further mechanistic exploration of CHD.

Of course, this study also has some shortcomings: the sample size is relatively small, and the metabolite differences between CHD subtypes could not be analyzed comparatively. The basic characteristics the of cases and controls are somewhat inconsistent due to the limited collection of samples in biobanks, which may interfere with the results. In addition, only internal data were used in the validation model, and no external database was used for verification. Future studies should focus on larger sample sizes for in-depth analysis and validation. This study will provide certain directions and ideas for future studies.

## Supplementary Information

**Additional file 1:** **Table S1.** Characteristics of the cases. **Table S2.** The list of 118 differential metabolites. **Figure S1.** The PCA and clustering heat maps of two groups. Fig. A, Cluster map of all samples. Fig. B, Repeated correlation assessment results. Fig. C The volcano map for differential metabolites screening. **Figure S2.** The out of bag error of random forest model with different number of variables contained in each decision tree. **Figure S3.** The out of bag error of random forest model with different number of decision tree. **Figure S4.** The confusion matrix for congenital heart defect.

## Data Availability

The data can be obtained by reasonable request from the corresponding author on reasonable requests.
