# Supplementary material for: Biomarkers for isolated congenital heart disease based on maternal amniotic fluid metabolomics analysis
Source: BMC Cardiovasc Disord. 2022 Nov 20;22:495. doi: 10.1186/s12872-022-02912-2 (PMC9677635; doi:10.1186/s12872-022-02912-2)
Supplement: Supplementary file 1 — Additional file 1: Table S1. Characteristics of the cases. Table S2. The list of 118 differential metabolites. Figure S1. The PCA and clustering heat maps of two groups. Fig. A, Cluster map of all samples. Fig. B, Repeated correlation assessment results. Fig. C The volcano map for differential metabolites screening. Figure S2. The out of bag error of random forest model with different number of variables contained in each decision tree. Figure S3. The out of bag error of random forest model with different number of decision tree. Figure S4. The confusion matrix for congenital heart defect. [file 12872_2022_2912_MOESM1_ESM.docx]

Appendix

**Biomarkers for Isolated Congenital Heart Disease Based on Maternal Amniotic Fluid Metabolomics analysis**

Xuelian Yuan, ^1,2*^, Lu Li, ^1,2*^, Hong Kang ^1,2^, Meixian Wang ^1,2^, Jing Zeng ^3^, Yanfang Lei ^4^, Nana Li, ^1,2^ Ping Yu, ^1,2^ Xiaohong Li, ^1,2§^ Zhen Liu ^1,2§^

1. **Characteristics of the cases**

Twenty-eight cases were recruited in this study. The characteristics were shown in the Table S1.

Table S1. Characteristics of the cases

| Name | Hospital | Age | Gestational  age | weight | hight | Gravidity | Fetal  gender | Diagnose |
| --- | --- | --- | --- | --- | --- | --- | --- | --- |
| case1 | 1 | 34 | 28 | 46 | 158 | 2 | 1 | Q20.4 |
| case2 | 1 | 31 | 21 | 50 | 162 | 2 | 1 | Q21.3 |
| case3 | 1 | 27 | 33 | 37 | 155 | 2 | 2 | Q21.0 |
| case4 | 1 | 32 | 36 | 52 | 167 | 2 | 1 | Q21.3 |
| case5 | 1 | 33 | 20 | 50 | 159 | 4 | 2 | Q21.0 |
| case6 | 1 | 20 | 36 | 65 | 156 | 2 | 2 | Q21.3 |
| case7 | 2 | 31 | 32 | 60 | 160 | 4 | 1 | Q20.4 |
| case8 | 2 | 28 | 23 | 58 | 170 | 2 | 1 | Q21.3 |
| case9 | 2 | 29 | 29 | 44 | 159 | 1 | 2 | Q21.0 |
| case10 | 1 | 22 | 27 | 57 | 171 | 1 | 1 | Q22.3;Q24.5 |
| case11 | 2 | 31 | 26 | 50 | 155 | 2 | 2 | Q20.6 |
| case12 | 2 | 26 | 16 | 54 | 158 | 1 | 2 | Q21.2 |
| case13 | 2 | 28 | 28 | 45 | 157 | 1 | 1 | Q21.3 |
| case14 | 2 | 22 | 29 | 51.5 | 159 | 1 | 2 | Q21.2;Q20.4 |
| case15 | 2 | 31 | 25 | 44 | 158 | 1 | 1 | Q20.4 |
| case16 | 2 | 35 | 17 | 55 | 151 | 7 | 2 | Q21.2 |
| case17 | 1 | 25 | 28 | 50 | 160 | 1 | 2 | Q21.0 |
| case18 | 1 | 25 | 25 | 47 | 156 | 2 | 1 | Q21.3 |
| case19 | 1 | 31 | 26 | 50 | 165 | 1 | 2 | Q21.2;Q20.4 |
| case20 | 3 | 23 | 28 | 49 | 162.5 | 1 | 1 | Q21.0 |
| case21 | 3 | 29 | 20 | 57 | 162 | 2 | 2 | Q21.3 |
| case22 | 3 | 27 | 31 | 70 | 162 | 3 | 2 | Q20.0 |
| case23 | 3 | 24 | 33 | 45 | 160 | 2 | 2 | Q21.0;Q20.3;Q25.5 |
| case24 | 3 | 35 | 28 | 66 | 157 | 4 | 2 | Q21.3 |
| case25 | 3 | 26 | 25 | 48 | 149 | 1 | 2 | Q21.3 |
| case26 | 3 | 28 | 29 | 45 | 154 | 2 | 2 | Q21.3 |
| case27 | 3 | 27 | 27 | 45 | 155 | 1 | 2 | Q21.0 |
| case28 | 3 | 24 | 31 | 42 | 160 | 2 | 2 | Q20.0 |

1. **Sample testing**

The clustering heat map analysis was performed on all samples (Figure S1A, B). After the screening condition of FC>2, P value<0.01 and VIP> 1.50, a total of 118 differential metabolites were screened shown as volcano map in Figure S1C. All 118 differential metabolites were listed in Table S2.

A B

C

Figure S1. The PCA and clustering heat maps of two groups

Fig. A, Cluster map of all samples. Fig. B, Repeated correlation assessment results. Fig. C the volcano map for differential metabolites screening.

Table S2 The list of 118 differential metabolites

| #ID | name | controls_mean | cases_mean | fold_change | log_2_FC | *P*-value | VIP | regulated |
| --- | --- | --- | --- | --- | --- | --- | --- | --- |
| meta_49 | Oryzalide B | 2.19E-05 | 2.79E-06 | 0.127365 | -9.21636 | 0.000262 | 1.540083 | down |
| meta_88 | (2S)-2-Butanol O-[b-D-Apiofuranosyl-(1->6)-b-D-glucopyranoside] | 2.89E-05 | 1.28E-05 | 0.443797 | -2.99459 | 0.000905 | 1.514872 | down |
| meta_91 | Androstenedione | 1.27E-05 | 2.36E-06 | 0.186506 | -4.49354 | 0.00025 | 1.558403 | down |
| meta_101 | 9alpha-(3-Methyl-2E-pentenoyloxy)-4S-hydroxy-10(14)-oplopen-3-one | 7.54E-05 | 2.51E-05 | 0.332942 | -1.26554 | 0.000162 | 1.582526 | down |
| meta_107 | 10-Acetoxy-8-heptadecene-4,6-diyn-3-ol | 1.33E-05 | 1.99E-06 | 0.149503 | -9.30444 | 0.000173 | 1.606162 | down |
| meta_132 | (4R,5S,7R,11x)-11,12-Dihydroxy-1(10)-spirovetiven-2-one 12-glucoside | 1.92E-05 | 3.03E-06 | 0.157493 | -7.11337 | 0.000136 | 1.621428 | down |
| meta_147 | N-2-[4-(3,3-Dimethylallyloxy)phenyl]ethylcinnamide | 1.53E-05 | 2.78E-06 | 0.182202 | -9.14098 | 2.15E-05 | 1.772857 | down |
| meta_202 | N-Oleoylethanolamine | 5.17E-05 | 5.82E-06 | 0.112608 | 0.51422 | 0.000137 | 1.577154 | down |
| meta_246 | 1-dodecanoyl-glycero-3-phosphate | 7.32E-06 | 5.27E-07 | 0.071949 | -12.7147 | 4.21E-05 | 1.763889 | down |
| meta_248 | O-Desmethylvenlafaxine glucuronide | 5.52E-06 | 1.53E-05 | 2.773801 | 6.393648 | 0.001463 | 1.631017 | up |
| meta_256 | 3beta-3-Hydroxy-18-lupen-21-one | 1.01E-05 | 1.9E-06 | 0.187549 | -7.87638 | 0.000185 | 1.623522 | down |
| meta_259 | MG(0:0/18:3(9Z,12Z,15Z)/0:0) | 8.99E-05 | 2.61E-05 | 0.290392 | -1.43397 | 0.000419 | 1.508556 | down |
| meta_288 | N-(1-Deoxy-1-fructosyl)phenylalanine | 6.91E-06 | 1.81E-05 | 2.618526 | 6.701262 | 0.003351 | 1.510552 | up |
| meta_308 | Austalide L | 7.7E-06 | 3.7E-05 | 4.80275 | 7.787309 | 0.000235 | 1.974888 | up |
| meta_332 | Kyotorphin | 2.47E-06 | 1.29E-05 | 5.213568 | 9.857466 | 0.000377 | 1.900975 | up |
| meta_350 | 3,6-Ditigloyloxytropan-7-ol | 2.7E-05 | 8.87E-05 | 3.287709 | 7.93729 | 0.000421 | 1.890892 | up |
| meta_361 | 16,17-Dihydro-16a,17-dihydroxygibberellin A4 17-glucoside | 1.37E-05 | 4.28E-07 | 0.031148 | -19.127 | 0.000172 | 1.609594 | down |
| meta_392 | Desglucocheirotoxin | 7.79E-06 | 7.93E-07 | 0.101765 | -16.0976 | 0.000331 | 1.505536 | down |
| meta_470 | alpha-Zearalenol | 6.21E-06 | 2.41E-05 | 3.876751 | 7.05244 | 7.64E-06 | 2.258061 | up |
| meta_505 | PE(18:0/16:0) | 1.07E-05 | 2.58E-07 | 0.024102 | -16.2034 | 0.000562 | 1.502833 | down |
| meta_581 | Citreoviridin D | 4.41E-06 | 1.73E-05 | 3.934493 | 12.60502 | 0.000156 | 1.932214 | up |
| meta_599 | 12-hydroxyicosanoic acid | 0.000243 | 0.000493 | 2.030786 | 1.29404 | 0.001377 | 1.687613 | up |
| meta_604 | (2S,4R,6S)-2-[2-(4-hydroxy-3-methoxyphenyl)ethyl]tetrahydro-6-(4-hydroxy-3,5-dimethoxyphenyl)-2H-pyran-4-ol | 1.07E-05 | 3.12E-05 | 2.913849 | 8.527035 | 0.001023 | 1.62649 | up |
| meta_631 | Lansic acid | 3.54E-05 | 4.41E-06 | 0.124531 | -19.4927 | 0.000186 | 1.591114 | down |
| meta_632 | Nomilinic acid | 7.54E-05 | 1.42E-05 | 0.187751 | -8.82229 | 0.000102 | 1.644382 | down |
| meta_638 | 17-Hydroxyprogesterone | 1.63E-05 | 2.85E-06 | 0.174477 | -2.76843 | 0.00019 | 1.580548 | down |
| meta_675 | 8-Propanoylneosolaniol | 1.63E-06 | 1.29E-05 | 7.865036 | 15.51352 | 0.000143 | 2.026943 | up |
| meta_686 | 11-Hydroxyprogesterone 11-glucuronide | 0.000111 | 1.76E-05 | 0.157604 | -2.85223 | 0.000323 | 1.51859 | down |
| meta_723 | PE(15:0/P-18:0) | 3.16E-05 | 2.81E-06 | 0.088924 | -15.1247 | 8.67E-05 | 1.703662 | down |
| meta_741 | 5-(2,3-Dihydroxy-3-methylbutyl)-4-(3,4-epoxy-4-methylpentanoyl)-3,4-dihydroxy-2-isopentanoyl-2-cyclopenten-1-one | 1.25E-05 | 1.19E-06 | 0.095285 | -9.33716 | 0.000435 | 1.505637 | down |
| meta_803 | 3,4-DHPEA-EA | 1.29E-06 | 5.14E-06 | 3.982744 | 13.60965 | 0.00162 | 1.628944 | up |
| meta_818 | 11-Dihydro-12-norneoquassin | 2.99E-06 | 1.11E-07 | 0.03732 | -18.1858 | 6.5E-05 | 1.692965 | down |
| meta_838 | 3-Methylglutarylcarnitine | 1.09E-05 | 2.83E-05 | 2.602892 | 2.316583 | 1.35E-05 | 2.103115 | up |
| meta_873 | 3-Hydroxynevirapine | 3.4E-06 | 1.03E-05 | 3.036043 | 6.240452 | 0.000271 | 1.789586 | up |
| meta_880 | Ketotifen-N-glucuronide | 1.56E-05 | 4.62E-07 | 0.029534 | -16.1991 | 0.000427 | 1.509915 | down |
| meta_881 | 12S-HHT | 1.59E-05 | 3.66E-06 | 0.230254 | -1.55234 | 0.000343 | 1.525549 | down |
| meta_888 | 1-Phenyl-1,3-heneicosanedione | 2.29E-06 | 6.79E-06 | 2.96821 | 10.8986 | 0.002204 | 1.515261 | up |
| meta_901 | 2,6-Di-tert-butylbenzoquinone | 1.76E-05 | 6.23E-06 | 0.353732 | -1.67827 | 1.35E-06 | 1.996661 | down |
| meta_904 | Alvimopan | 7.66E-06 | 4.16E-05 | 5.425741 | 12.20972 | 5.01E-06 | 2.298024 | up |
| meta_959 | Lycopersiconol | 3.07E-05 | 3.68E-06 | 0.119566 | -2.63935 | 0.00026 | 1.55784 | down |
| meta_968 | Argatroban | 0.000292 | 7.41E-05 | 0.25331 | -3.04826 | 0.00033 | 1.528371 | down |
| meta_974 | Citronellyl anthranilate | 5.74E-06 | 3.42E-07 | 0.059585 | -15.1221 | 0.000259 | 1.560444 | down |
| meta_984 | Ergotamine | 7.62E-05 | 0.000204 | 2.671441 | 0.180657 | 0.00296 | 1.640044 | up |
| meta_1011 | Gluten exorphin B5 | 4.43E-06 | 1.27E-05 | 2.861935 | 7.114138 | 0.000332 | 1.804946 | up |
| meta_1024 | Mytilin B | 1.53E-05 | 4.41E-05 | 2.893657 | 5.172277 | 3.45E-05 | 2.07986 | up |
| meta_1054 | Norfluoxetine glucuronide | 8.02E-06 | 3.79E-07 | 0.047292 | -14.6499 | 0.000559 | 1.535488 | down |
| meta_1058 | Valganciclovir | 6.28E-06 | 2.01E-05 | 3.198633 | 5.980663 | 6.68E-05 | 2.019678 | up |
| meta_1083 | Epoxyfumitremorgin C | 2.99E-05 | 7.71E-05 | 2.575619 | 4.613393 | 0.00017 | 1.747099 | up |
| meta_1104 | (3beta,5alpha,6beta,22E,24R)-23-Methylergosta-7,22-diene-3,5,6-triol | 1.69E-07 | 3.4E-06 | 20.06003 | 12.74613 | 0.004658 | 1.569122 | up |
| meta_1164 | Ethyl 3-hydroxyoctanoate O-[glucosyl-(1->6)-glucoside] | 2.83E-05 | 1.76E-06 | 0.062219 | -15.0513 | 0.000345 | 1.522166 | down |
| meta_1173 | Cyclomammeisin | 1.45E-05 | 3.32E-05 | 2.285937 | 5.143485 | 0.000461 | 1.67806 | up |
| meta_1195 | N-[(4E,8Z)-1,3-dihydroxyoctadeca-4,8-dien-2-yl]hexadecanamide 1-glucoside | 0.000118 | 5.6E-05 | 0.47292 | -1.22454 | 5.47E-08 | 2.296768 | down |
| meta_1197 | Marmeline | 3.95E-05 | 8.51E-05 | 2.15552 | 3.931791 | 0.001357 | 1.618118 | up |
| meta_1204 | PE(15:0/16:0) | 0.001457 | 8.57E-05 | 0.058825 | -2.32964 | 0.000417 | 1.53948 | down |
| meta_1250 | Cadabicine methyl ether | 5.38E-06 | 1.51E-05 | 2.809177 | 6.152306 | 0.000993 | 1.656339 | up |
| meta_1261 | Glabrolide | 1.05E-05 | 3.12E-06 | 0.296532 | -0.42436 | 1.9E-06 | 2.017402 | down |
| meta_1268 | Kuwanon A | 5.44E-06 | 3.03E-05 | 5.564739 | 8.690542 | 0.00071 | 1.830792 | up |
| meta_1325 | Methyl 7-epi-12-hydroxyjasmonate glucoside | 2.46E-05 | 3.47E-06 | 0.140854 | -7.11096 | 0.000502 | 1.504111 | down |
| meta_1327 | Sporotrichiol | 6.61E-06 | 1.71E-06 | 0.258882 | -10.5559 | 0.000268 | 1.53248 | down |
| meta_1332 | Corchorusoside A | 1.58E-06 | 6.77E-06 | 4.279065 | 12.78294 | 0.00056 | 1.7806 | up |
| meta_1341 | NADH | 1.02E-05 | 3.44E-05 | 3.383956 | 5.86185 | 8.8E-06 | 2.186495 | up |
| meta_1354 | LysoPC(10:0) | 9.21E-06 | 2.87E-06 | 0.31205 | -1.32887 | 4.78E-05 | 1.803123 | down |
| meta_1355 | Dukunolide B | 9.41E-05 | 1.17E-05 | 0.124238 | -10.2082 | 0.000608 | 1.505983 | down |
| meta_1372 | Adenosine triphosphate | 2.69E-06 | 9.07E-06 | 3.373075 | 11.57749 | 0.002362 | 1.603385 | up |
| meta_1406 | cis-Annonacin-10-one | 0.000566 | 5.18E-05 | 0.091555 | -1.90608 | 0.000591 | 1.509595 | down |
| meta_1417 | 31-Hydroxy rifabutin | 9.37E-06 | 2.41E-05 | 2.575783 | 4.875001 | 0.001949 | 1.620666 | up |
| meta_1431 | Agnuside | 5.01E-05 | 4.3E-06 | 0.085833 | -9.09059 | 0.000297 | 1.540056 | down |
| meta_1461 | PE(MonoMe(11,5)/MonoMe(13,5)) | 0.001452 | 0.000527 | 0.362865 | -1.33526 | 1.13E-06 | 2.084292 | down |
| meta_1475 | Schidigeragenin C | 0.006121 | 0.002693 | 0.439922 | -0.83846 | 0.000206 | 1.605491 | down |
| meta_1489 | Rifaximin | 6.41E-07 | 7.73E-06 | 12.05882 | 17.38928 | 0.00021 | 1.991036 | up |
| meta_1490 | (S)-Hydroxyoctanoyl-CoA | 2.51E-07 | 6.35E-06 | 25.32421 | 15.6811 | 0.004034 | 1.626502 | up |
| meta_1492 | Valproic acid glucuronide | 1.52E-05 | 1.87E-06 | 0.122923 | -6.3983 | 8.69E-05 | 1.693787 | down |
| meta_1494 | Aeglin | 3.61E-05 | 4.65E-06 | 0.12897 | -8.62822 | 0.000408 | 1.505862 | down |
| meta_1502 | Cryptomeridiol 11-rhamnoside | 2.36E-05 | 2.71E-06 | 0.114793 | -7.58007 | 0.000272 | 1.556573 | down |
| meta_1512 | Lansioside B | 6.36E-05 | 1.35E-05 | 0.211653 | -1.6454 | 7.15E-05 | 1.717834 | down |
| meta_1555 | 6-Oxocyclohex-1-ene-1-carboxyl-CoA | 3.98E-06 | 1.17E-05 | 2.954261 | 12.19812 | 0.000108 | 1.805115 | up |
| meta_1562 | 1,2-Dihydrodehydroguaiaretic acid | 7.65E-06 | 2.25E-05 | 2.939248 | 6.58186 | 0.000552 | 1.764695 | up |
| meta_1577 | PC(24:0/P-18:0) | 4.43E-06 | 1.24E-05 | 2.801694 | 7.233433 | 0.001197 | 1.627734 | up |
| meta_1587 | 4-[N-(p-Coumaroyl)serotonin-4''-yl]-N-feruloylserotonin | 7.13E-05 | 0.000236 | 3.314596 | 2.248907 | 6.44E-07 | 2.388946 | up |
| meta_1612 | Everolimus | 1.94E-05 | 4.05E-05 | 2.093015 | 2.954394 | 0.001387 | 1.638871 | up |
| meta_1648 | Cytochalasin Opho | 7.46E-05 | 2.6E-05 | 0.348971 | -1.19044 | 0.00033 | 1.583791 | down |
| meta_1725 | Dolichyl b-D-glucosyl phosphate | 7.01E-06 | 2.6E-05 | 3.710983 | 10.09642 | 2.55E-05 | 2.11502 | up |
| meta_1726 | NADP | 6.97E-06 | 1.68E-05 | 2.411128 | 6.146356 | 0.002249 | 1.551582 | up |
| meta_1734 | Mocimycin | 4.39E-06 | 1.26E-05 | 2.866185 | 6.209673 | 0.00035 | 1.84011 | up |
| meta_1753 | LPA(0:0/18:1(9Z)) | 2.87E-05 | 1.13E-05 | 0.394072 | -0.92322 | 0.00031 | 1.61394 | down |
| meta_1813 | PC(o-14:0/16:1(9Z)) | 0.006308 | 0.00117 | 0.185429 | -1.46996 | 0.000507 | 1.51687 | down |
| meta_1820 | Neoacrimarine G | 4.97E-05 | 4.6E-06 | 0.092643 | -10.8779 | 0.00043 | 1.544501 | down |
| meta_1849 | PIP2(16:0/20:1(11Z)) | 9.02E-07 | 3.4E-06 | 3.771044 | 6.814973 | 0.002132 | 1.707115 | up |
| meta_1871 | Cefodizime | 1.33E-05 | 3.16E-05 | 2.378392 | 5.867671 | 3.93E-05 | 1.803397 | up |
| meta_1915 | 1-(3,4-Dimethoxyphenyl)-1,2-ethanediol 2-O-b-D-glucoside | 7.75E-06 | 2.05E-05 | 2.640664 | 6.690741 | 0.000104 | 1.837951 | up |
| meta_1949 | Citreoviridin A | 5.22E-05 | 8.29E-06 | 0.158753 | -0.30958 | 0.000265 | 1.538261 | down |
| meta_1965 | Flavidulol D | 8.01E-05 | 6.34E-06 | 0.079234 | -5.53499 | 0.000204 | 1.580824 | down |
| meta_1970 | Dulcoside A | 4.06E-06 | 1.12E-05 | 2.769505 | 7.726882 | 0.00254 | 1.601109 | up |
| meta_1973 | Lysyl-Valine | 7.13E-06 | 1.1E-06 | 0.153788 | -9.99452 | 0.000287 | 1.562876 | down |
| meta_1980 | Didemethylcitalopram | 1.23E-05 | 5.16E-05 | 4.188054 | 12.85737 | 0.000786 | 1.826426 | up |
| meta_1988 | Elaterinide | 5.45E-06 | 1.54E-05 | 2.829273 | 8.936301 | 0.00064 | 1.749812 | up |
| meta_2009 | Ticarcillin | 5.62E-06 | 1.28E-05 | 2.277204 | 5.736405 | 1.27E-05 | 1.895334 | up |
| meta_2010 | Deoxycorticosterone | 4.1E-05 | 8E-06 | 0.195437 | -3.24698 | 0.000395 | 1.505331 | down |
| meta_2031 | 2-(Arabinosylamino)-3-(glucosylamino)propanenitrile | 3.83E-06 | 1.31E-05 | 3.416066 | 10.4306 | 2.72E-05 | 2.086207 | up |
| meta_2036 | Cyclizine | 0.000155 | 5.28E-05 | 0.340937 | -1.41873 | 0.000536 | 1.520226 | down |
| meta_2066 | CerP(d18:1/26:1(17Z)) | 1.96E-05 | 4.86E-05 | 2.486943 | 9.500554 | 0.000778 | 1.600702 | up |
| meta_2067 | Honyudisin | 6E-06 | 2.11E-05 | 3.523045 | 10.70845 | 0.00013 | 1.965081 | up |
| meta_2095 | Artocarpin | 1.51E-06 | 6.15E-06 | 4.065879 | 10.80783 | 0.000398 | 1.88511 | up |
| meta_2132 | Hv-NCC-1 | 2.61E-06 | 7.53E-06 | 2.882154 | 8.700627 | 0.000182 | 2.008864 | up |
| meta_2134 | Soyasapogenol C | 3.51E-05 | 5.67E-06 | 0.161414 | -5.15651 | 3.25E-05 | 1.764927 | down |
| meta_2137 | Medicagenic acid 28-O-[b-D-xylosyl-(1->4)-a-L-rhamnosyl-(1->2)-a-L-arabinosyl] ester | 1.3E-05 | 3.04E-05 | 2.3388 | 3.036875 | 7.13E-05 | 1.977643 | up |
| meta_2147 | CD 1790 | 1.2E-05 | 3.14E-05 | 2.614196 | 0.256617 | 0.000634 | 1.727 | up |
| meta_2194 | 7b-Hydroxy-3-oxo-5b-cholanoic acid | 4.17E-05 | 2.61E-06 | 0.062577 | -14.2573 | 0.000313 | 1.555435 | down |
| meta_2220 | 4-Nitrophenyl sulfate | 1.83E-05 | 4.9E-05 | 2.680695 | 4.576541 | 0.000378 | 1.690604 | up |
| meta_2232 | Physagulin G | 7.41E-07 | 4.41E-06 | 5.957915 | 14.42771 | 0.000345 | 1.940405 | up |
| meta_2284 | 5-Aminopentanamide | 0.001276 | 0.000349 | 0.273662 | -1.35874 | 2.94E-05 | 1.758329 | down |
| meta_2313 | (-)-Epicatechin sulfate | 7.97E-07 | 4.47E-06 | 5.607414 | 14.78583 | 5.46E-05 | 2.139213 | up |
| meta_2327 | Calamendiol | 3.57E-05 | 5.28E-06 | 0.148018 | -4.4779 | 0.000551 | 1.501347 | down |
| meta_2347 | Gibberellin A4 glucosyl ester | 2.05E-06 | 8.67E-06 | 4.223715 | 13.074 | 2.63E-05 | 2.138869 | up |
| meta_2362 | (1S,2S,4R,8S)-p-Menthane-1,2,8,9-tetrol 2-glucoside | 7.66E-06 | 2.31E-05 | 3.017848 | 8.442674 | 3.93E-05 | 2.029685 | up |
| meta_2373 | SM(d18:0/16:1(9Z)(OH)) | 0.00073 | 0.000205 | 0.280456 | 0.187917 | 1.4E-06 | 2.033504 | down |
| meta_2385 | 11-Dehydro-thromboxane B2 | 4.39E-05 | 6.62E-06 | 0.150921 | -1.54265 | 0.000686 | 1.523558 | down |
| meta_2468 | Bebeerine | 8.82E-06 | 4.57E-05 | 5.177571 | 5.125051 | 0.00466 | 1.622466 | up |

1. **Random Forest Algorithm**

The out of bag error of random forest could be seen in Figure S2,S3.


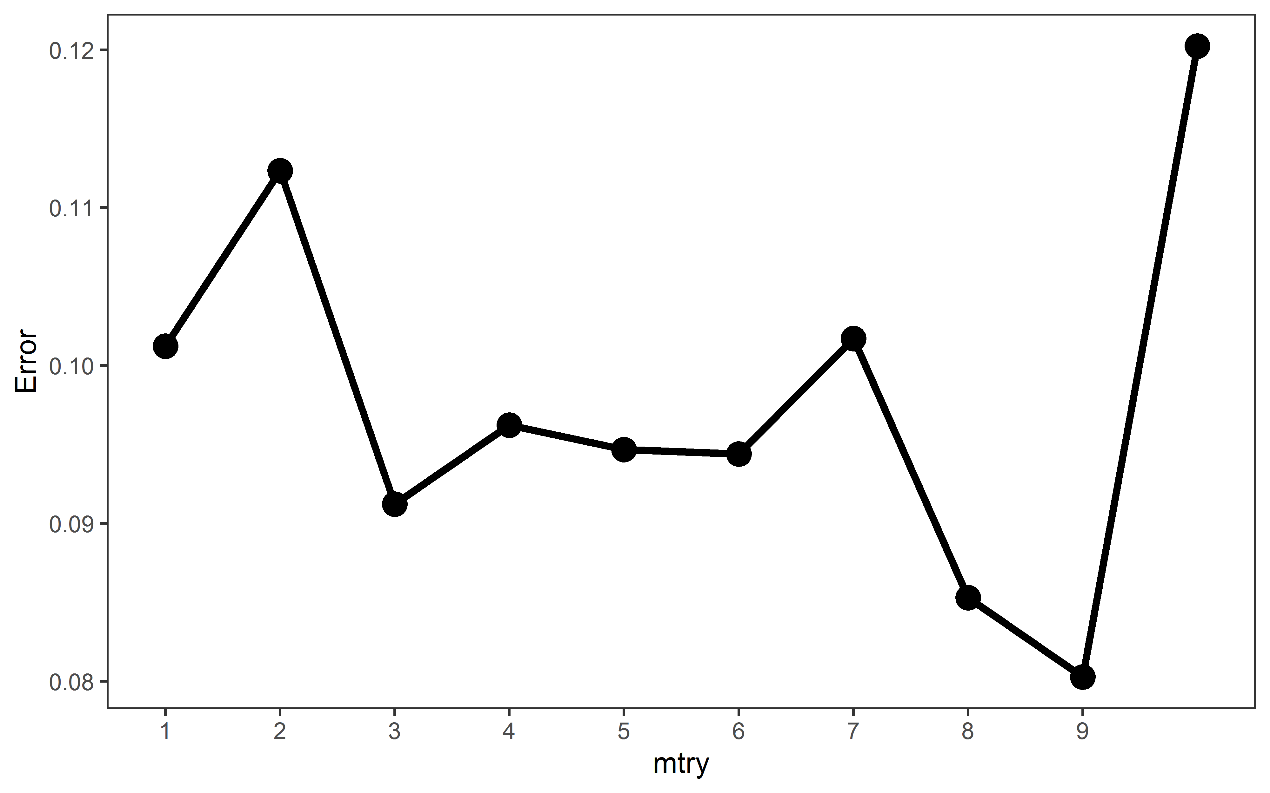


Figure S2. The out of bag error of random forest model with different number of variables contained in each decision tree.


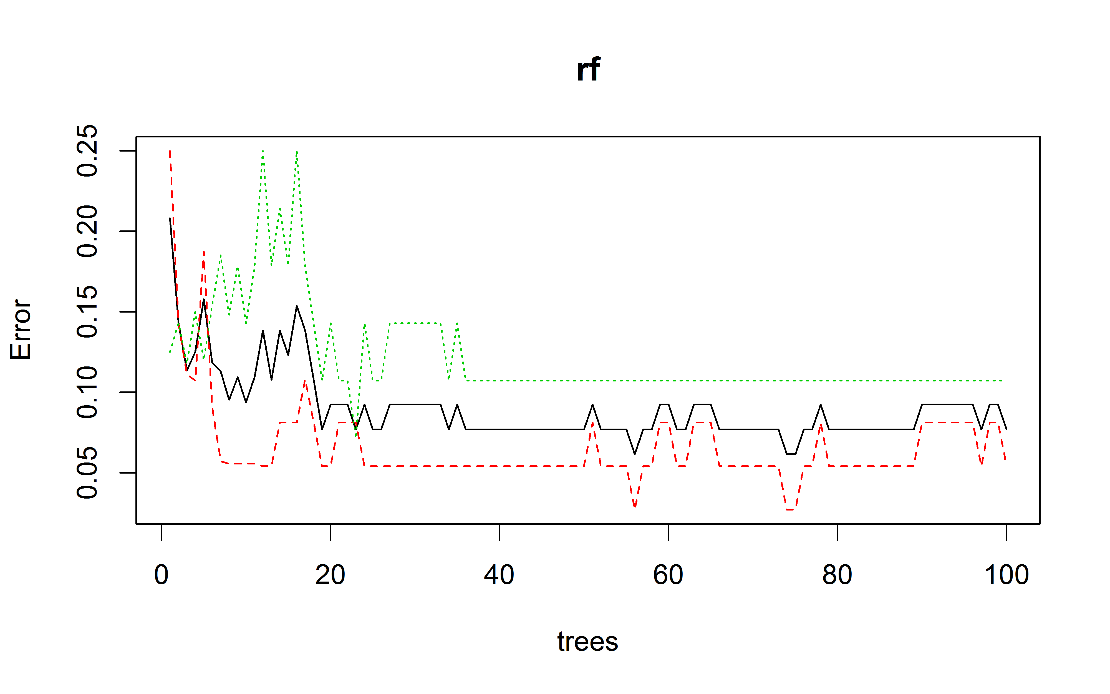


Figure S3. The out of bag error of random forest model with different number of decision tree.

The performance of the combination of these markers was assessed by logistic regression model for predicting the class of the subjects, and the Akaike Information criterion (AIC) and Bayesian Information criterion (BIC) were 25.15 and 33.85, respectively. The confusion matrix of the logistic regression model for predicting the class of the subjects is shown in Fig. 3. In binary classification, the PPV and NPV are the percentage of correctly classified compounds among all subjects predicted as positives or negatives, respectively. The PPV and NPV of the model were 96.3% and 94.7%, respectively. The calculation of the Positive Predictive Value (PPV), Equation, and Negative Predictive Value (NPV), Equation, is shown below:

$$PPV= \frac{True Positives}{True Positives+False Positives}$$

$$PPV= \frac{26}{26+1}=96.3\%$$

$$NPV= \frac{True Negatives}{True Negatives+False Negatives}$$

$$PPV= \frac{36}{36+2}=94.7\%$$

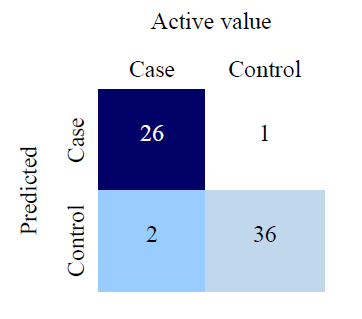


Figure S4. The confusion matrix for congenital heart defect.
